# A case report of a colouterine fistula due to sigmoid diverticulitis

**DOI:** 10.1093/jscr/rjae596

**Published:** 2024-09-23

**Authors:** Catherine L A Chang, Kenley R Unruh, Chad E Cragle, Ravi Moonka

**Affiliations:** Department of General Surgery, Virginia Mason Medical Center, Ninth and Seneca Street, Seattle, WA 98101, United States; Department of General Surgery, Virginia Mason Medical Center, Ninth and Seneca Street, Seattle, WA 98101, United States; Department of General Surgery, Virginia Mason Medical Center, Ninth and Seneca Street, Seattle, WA 98101, United States; Department of General Surgery, Virginia Mason Medical Center, Ninth and Seneca Street, Seattle, WA 98101, United States

**Keywords:** colouterine fistula, diverticular fistula, diverticulitis, colon, uterus, case report

## Abstract

Colouterine fistulas are generally seen in post-menopausal patients and present with abdominal pain and non-physiologic vaginal drainage. A history of uterine pathology or diverticulitis is generally lacking. Visualization of the passage of contrast from the gastrointestinal tract to the uterus is not necessary to make the diagnosis. We present the case of a 44-year-old woman successfully treated for a colouterine fistula due to sigmoid diverticulitis. A variety of surgical approaches have been described to correct this fistula, and a minimally invasive colectomy without ileostomy or colostomy appears to be a safe approach.

## Introduction

Sigmoid diverticulitis can result in fistula formation in any organ in the pelvis. Colouterine fistulas constitute only 3% of such connections and are rare compared to colovesicular (65%), colovaginal (25%), and coloenteric (7%) disease [1]. The relatively thick myometrium is presumed to protect the uterus from an eroding infection. This infrequent occurrence can complicate standard diagnostic and therapeutic maneuvers. We describe the management of a younger patient found to have a colouterine fistula and compare her presentation and management to that of previously reported cases.

## Case report

A 44-year-old woman presented to our emergency department with severe abdominal pain and malodorous, feculent-appearing vaginal drainage. The patient had a 3-year history of intermittent abdominal pain, but never as intractable as this presentation. She had undergone global thermal endometrial ablation for dysfunctional uterine bleeding 3 years prior. She had long-standing type II diabetes treated with glipizide and metformin. On exam, the patient’s body mass index was 41.6 kg/m^2^. Her temperature was 39.4°C. Her abdomen was diffusely tender on palpation.

**Figure 1:**
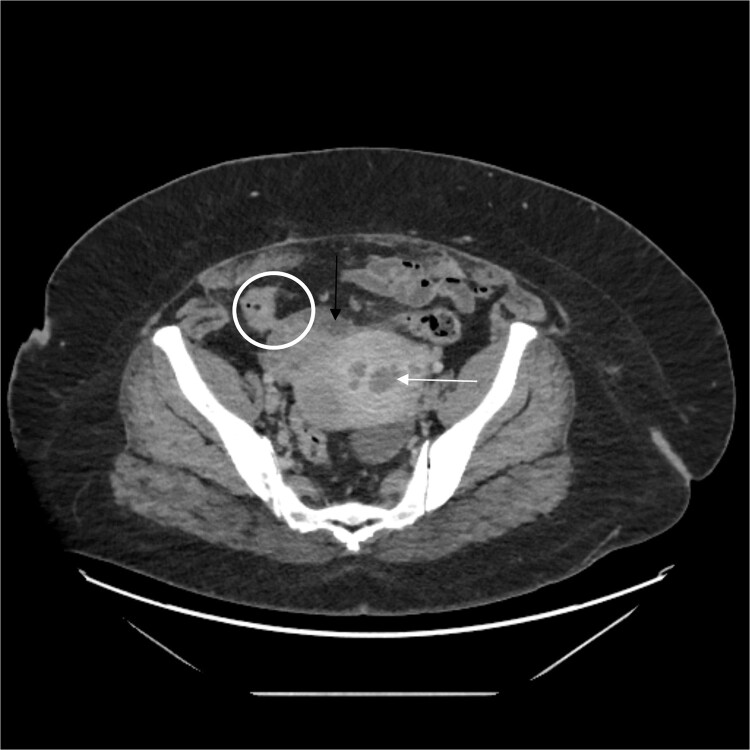
White arrow shows fluid in the uterus, black arrow shows phlegmon, and circle demonstrates colon and fistula.

A complete blood count was normal. CT imaging demonstrated fluid and air in the uterus, associated with a phlegmon that communicated with the sigmoid colon (Fig. 1). An ovarian cyst was incidentally noted posterior to the uterus.

On colonoscopy, diverticula were seen within the descending and sigmoid colon, but no malignancy nor inflammation. An endometrial biopsy demonstrated a fragmented endometrial polyp and fecal material but no malignancy.

The patient’s fevers and pain resolved with a course of intravenous and oral antibiotics. She was placed on a very low carbohydrate diet, resulting in a 55-pound weight loss and a drop in her hemoglobin A1c from 6.9% to 5.6%.

Three months following her initial presentation, the patient underwent a robotic-assisted laparoscopic sigmoid colectomy, hysterectomy, bilateral salpingectomy, and right oophorectomy. Bilateral retrograde indocyanine green ureteral injection facilitated intraoperative ureteral visualization. Cystoscopy demonstrated that the bladder was not injured during the hysterectomy. Feculent cervical discharge was seen during placement of the uterine manipulator. The sigmoid colon was inflamed and densely adherent to the body of the uterus; the uterus was bulky and mildly hyperemic. The sigmoid colon and the uterus were extracted through the vagina, obviating the need for a Pfannenstiel incision. The coloproctostomy was constructed in an end-to-end fashion using a 29 mm EEA stapler.

**Figure 2:**
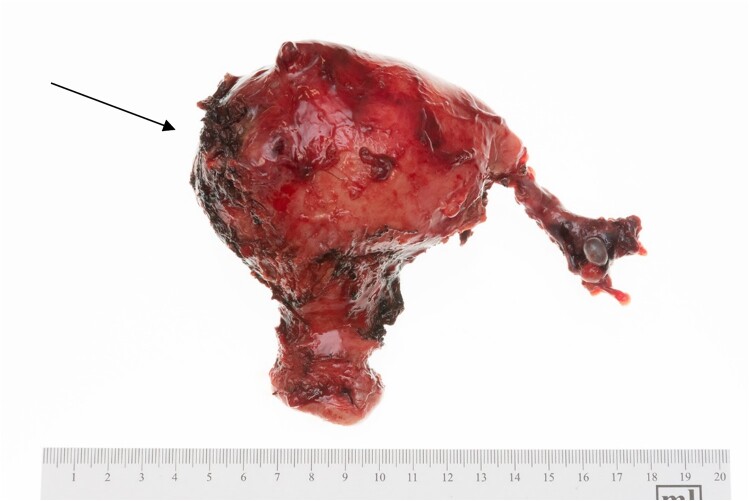
Uterus, cervix, and fallopian tube. The arrow marks the area of fistula formation.

**Figure 3:**
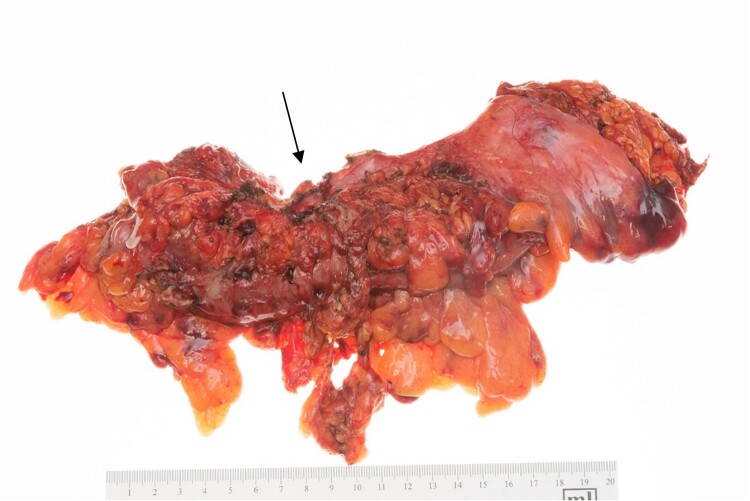
Sigmoid colon with arrow marking the area of fistula formation.

Final pathology results showed a grossly evident fistula tract measuring 1.7 cm in length in the lower anterior uterine segment extending through the myometrium and into the endometrial cavity (Fig. 2). This was accompanied by a benign leiomyoma with inflammation and a proliferative-type endometrium. Her cervix and bilateral fallopian tubes were normal. The sigmoid colon showed gross areas of perforation and diverticulosis (Fig. 3). Two areas of perforation were identified, 3.0 and 12.0 cm from the proximal surgical margin.

She recovered uneventfully and was discharged 3 days after surgery.

## Discussion

We performed a literature review using PubMed with the search terms ‘diverticulitis’ and ‘colouterine fistula’. We evaluated all 37 previously reported cases since 1957, described either in abstract or full manuscript form (Table 1). Abdominal pain and vaginal drainage were the most frequent symptoms noted in 21 and 23 of the 37 patients. Vaginal drainage was generally malodorous or clearly feculent. Three patients described vaginal flatulence. Nine patients were febrile. Our patient’s age of 44 was atypical, since only 2 of the 37 previous patients were under 60. The average age at presentation was 71 years.

**Table 1:** Characteristics and management of patients with colouterine fistula secondary to diverticulosis.

**Author**	**Year**	**Age**	**Symptoms**	**Diagnostic studies**	**Operation**	**Complication**	**Other**
Silva C, *et al.*	2024	71	None of common symptoms	CT, colonoscopy, TVUS, hysteroscopy, and biopsy	Open sigmoidectomy, total hysterectomy and bilateral salpingo-oophorectomy		
Aslam A, *et al.*	2024	74	Abdominal pain	CT	Open Hartmann w/ total hysterectomy and bilateral salpingo-oophorectomy	Post op ileus	Abscess noted
Anthony P, *et al.*	2023	68	None of common symptoms	CT	Open sigmoidectomy and subtotal hysterectomy		Abscess noted
Ismail I B, *et al.*	2021	67	Abdominal pain, vaginal discharge	CT	Laparoscopic sigmoidectomy w/ primary anastamosis		
Jha P, *et al.*	2021	28	Abdominal pain, vaginal discharge, fecaloid flow, gas passage through introitus		Total hysterectomy		Gossypiboma. Abscess noted
Valverde F, *et al.*	2020	82	Abdominal pain	CT	Abscess drainage (×2)		Spontaneous resoluation w/ IV abx
Pontone S, *et al.*	2021	62	Vaginal discharge, fecaloid flow, gas passage through introitus	Colonoscopy, gastrografin seen in uterus		Endoscopic self-exapandable metallic stent	
Galanis I, *et al.*	2021	74	Vaginal discharge	CT	Open total hysterectomy with partial jejunojejunostomy and partial sigmoidectomy bilateral salpingoophorectomy w/ primary anastamosis	Post op UTI	
Perez A, *et al.*	2021	70	Abdominal pain, vaginal discharge	CT, TVUS	Open Hartmann w/ total hysterectomy and bilateral salpingo-oophorectomy		
Bowker B	2020	80	Abdominal pain, fever	CT	Hartmann w/ unilateral oophorectomy		
Guevara Morales, G.R.	2018	67	Abdominal pain, fever	CT	Sigmoidectomy, total hysterectomy with bilateral salpingo-oophorectomy		
Aggarwal R, *et al*	2018	77	abdominal pain, vaginal discharge,fever	CT	sigmoidectomy, total hysterectomy and abscess drainage		Abscess noted w/ drain attempt
Aggarwal R, *et al.*	2018	73	Abdominal pain,vaginal discharge	CT	Sigmoidectomy, total hysterectomy		
Arakawa S, *et al*.	2017	74	Abdominal pain, malodorous vaginal discharge, fecaloid flow	CT, gastrografin seen in uterus	Laparoscopic sigmoidectomy, total hysterectomy	Vaginal stump dehiscence	Abscess noted
Banky B, *et al.*	2016	70	Vaginal discharge, fecaloid flow, gas passage through introitus	CT, colonoscopy	Laparoscopic sigmoidectomy w/ primary anastamosis		Colon adenocarcinoma Abscess noted w/ drain attempt
Tracy L, *et al.*	2016	66	Vaginal discharge	Unspecified	Unspecified		
Bermello Meza C, *et al.*	2015	77	Abdominal pain	Gastrografin seen in uterus			
Uzan J, *et al*.	2014	34	Abdominal pain, vaginal discharge, fecaloid flow, fever	CT	Open Hartmann partial sigmoidectomy and abscess drainage		Abscess noted w/ drain attempt
Pankaja S, *et al.*	2014	74	Abdominal pain, vaginal discharge	CT, colonoscopy, hysteroscopy and biopsy	Open Hartmann w/ total hysterectomy and bilateral salpingo-oophorectomy		
Maamer AB, *et al.*	2013	65	Abdominal pain, vaginal discharge, fecaloid flow, fever	CT, gastrografin	Open total hysterectomy w/ bilateral salpingo-oophorectomy		
Choi PW	2012	81	Abdominal pain, vaginal discharge	CT, colonoscopy	Sigmoidectomy		Abscess noted w/ drain attempt
Mandato VD, *et al.*	2012	62	Vaginal discharge, fecaloid flow	CT, colonoscopy, TVUS, gastrografin seen in uterus, hysteroscopy, and biopsy	Open sigmoidectomy, total hysterectomy w/ bilateral salpingo-oophorectomy		Abscess noted w/ drain attempt
Vilallonga R, *et al.*	2008	76	Abdominal pain, vaginal discharge	CT	Open Hartmann		
Kassab A, *et al.*	2008	78	None of common symptoms	Colonoscopy, TVUS, hysteroscopy and biopsy	Open total hysterectomy w/ bilateral salpingo-oophorectomy		Abscess noted w/ drain attempt
Trastour C, *et al.*	2006	70	Vaginal discharge	CT	Open left hemicolectomy		
Vijayaraghavan S	2006	70	Vaginal discharge	TVUS	Open sigmoidectomy		
Hoekstra A, *et al.*	2005	92	Abdominal pain	Unspecified	Sigmoidectomy and total hysterectomy		
Hoekstra A, *et al.*	2005	87	abdominal pain	Unspecified	sigmoidectomy and total hysterectomy		
Petignat P, *et al.*	2004	88	Vaginal discharge	CT, TAUS, gastrografin	Open sigmoidectomy, total hysterectomy w/ bilateral salpingo-oophorectomy		
Takada T, *et al.*	2004	69	None of common symptoms	Colonoscopy, gastrografin seen in uterus	Open partial sigmoidectomy, total hysterectomy w/ bilateral salpingo-oophorectomy		
Sentilhes L, *et al.*	2003	76	Abdominal pain, vaginal discharge w/ fecaloid flow and fever	CT, TVUS, gastrografin	Open sigmoidectomy, total hysterectomy w/ bilateral salpingo-oophorectomy		
Lal S, *et al.*	2002	67	Abdominal pain, fever	CT	Hartmann w/ sigmoidectomy, total hysterectomy and bilateral salpingo-oophorectomy		
Huettner PC	1992	69	Vaginal discharge	Gastrografin seen in uterus	Total hysterectomy		
Chaikof E, *et al.*	1984	70	Vaginal discharge, fecaloid flow	Barium enema	Sigmoidectomy		
Chaikof E, *et al.*	1984	66	Fever	CT	Hartmann, sigmoidectomy and hysterectomy		Abscess noted
McGregor RA, *et al.*	1960	62	Abdominal pain, vaginal discharge, fever	barium enema, X-ray	Sigmoidectomy, hysterectomy, bilateral salpingo-oophorectomy		Abscess noted w/ drain attempt
Smalley MA, *et al.*	1957	76	Fecaloid flow	Barium enema, hysterosalpingogram	Fistula resection w/ closure of openings on sigmoid colon and uterus		

One patient had adenocarcinoma of the colon, and another had a retained sponge from a prior operation, which played a role in the fistula formation. In most reports, diverticulitis was the presumed etiology. Fistula formation <6 months after ablation is rare [2, 3], so our patient’s endometrial ablation 3 years prior is not likely to have directly caused fistula formation but may have increased susceptibility. Our concern for malignancy led to a preoperative colonoscopy and endometrial biopsy, but these maneuvers were performed in only eight and four of the previous cases. CT imaging was performed in 24 of 37 patients. Seven patients had sonography (six transvaginal, one transabdominal). Of the nine patients who received gastrografin either per os or by enema, contrast was seen in the uterus of six.

Surgery need not be emergent. Despite our patient’s abdominal pain and fever, we were able to perform elective surgery after a 3-month period of prehabilitation, during which she lost weight and decreased her hemoglobin A1c. Of the 13 prior cases in which an abscess was noted, eight patients underwent an image-guided drainage procedure, and in one, the drain and antibiotics led to resolution of the fistula.

Three patients had laparoscopic surgery, and the remainder had open surgery. Eight patients underwent a Hartmann’s procedure with an end colostomy and closure of the rectum, and the remainder had a primary anastomosis. Only 23 patients had a hysterectomy, suggesting removal of the uterus is not mandatory when the colon is the culprit organ. Fourteen patients had a bilateral oophorectomy and one had a unilateral oophorectomy. In our patient, one ovary was removed due to a large cyst and involvement in the inflammation around the fistula, and the other ovary was left *in situ* to prevent systemic symptoms of menopause. Our patient was the first published case report of a colouterine fistula to be treated with a robotic sigmoid colectomy and hysterectomy with vaginal extraction and an intracorporeal anastomosis without the need of an extraction incision, but prior reports have established the safety of a minimally invasive approach and of a primary anastomosis. Complications in the literature were minimal and included a postoperative UTI, a postoperative ileus, and one vaginal cuff dehiscence.

Colouterine fistulas are uncommon, generally nonmalignant, and can usually be diagnosed by CT imaging. Not all patients undergo colonoscopy or endometrial biopsy. Surgical approaches vary, but almost always include a sigmoid colectomy. Our patient is the first to be treated with robot-assisted laparoscopic sigmoid colectomy and hysterectomy with vaginal extraction and an intracorporeal anastomosis, showing the feasibility of this advantageous approach.
